# Effects of a digital repetitive dietary education intervention on dietary knowledge, dietary self-care behaviors, and physiological indicators among hemodialysis patients in rural Korea: a quasi-experimental study

**DOI:** 10.7586/jkbns.26.005

**Published:** 2026-05-29

**Authors:** Eun-Ho Ha, Sun-Ju Park

**Affiliations:** 1 Department of Nursing, Jungwon University, Goesan-gun, Korea; 2 Hemodialysis Unit, Goesan Seongmo Hospital, Goesan-gun, Korea

**Keywords:** Biomarkers, Computer-assisted instruction, Diet therapy, Renal dialysis, Rural population

## Abstract

**Purpose:**

Digital-enhanced repetitive learning has been proposed as an effective strategy for improving dietary education among patients undergoing hemodialysis. This study examined the effects of a digital repetitive dietary education (DRDE) program on dietary knowledge, dietary self-care behaviors, and physiological indicators among hemodialysis patients residing in a rural area of South Korea.

**Methods:**

A quasi-experimental study with a nonequivalent control group and repeated measures was conducted. A total of 40 hemodialysis patients were recruited from a hemodialysis unit in a rural area of Chungcheongbuk-do, South Korea, and assigned to either an experimental group (n = 20) or a control group (n = 20). The experimental group participated in an 8-session DRDE program, whereas the control group received usual care. Data were analyzed using SPSS version 22.0 with the independent t-test, chi-square test, and 2 × 2 repeated-measures analysis of variance.

**Results:**

Participants in the experimental group demonstrated significantly greater improvements in dietary knowledge (F = 14.83, *p* = .001) and dietary self-care practice behaviors (F = 4.21, *p* = .048) compared with the control group. Significant improvements were also observed in selected physiological indicators, including interdialytic weight gain (F = 7.02, *p* = .012), serum potassium levels (F = 9.55, *p* = .004), and serum phosphorus levels (F = 4.62, *p* = .038).

**Conclusion:**

The DRDE program improved dietary knowledge, dietary self-care behaviors, and selected physiological outcomes among hemodialysis patients in rural areas. These findings suggest that the DRDE program is a practical and feasible nursing intervention for dietary management in rural hemodialysis settings with limited educational resources.

## INTRODUCTION

### 1. Background

Rapid population aging in Korea has contributed to a growing prevalence of chronic diseases [1]. Among these, chronic kidney disease is a major condition that necessitates renal replacement therapy for survival. According to the 2024 Korean Renal Data System Annual Report published by the Korean Society of Nephrology (KSN), 117,933 patients (84.6%) were receiving hemodialysis in 2023, and individuals aged 65 years or older accounted for 59.8% of this population, indicating a pronounced aging trend among patients undergoing dialysis [2]. These demographic shifts underscore the increasing importance of structured educational interventions aimed at enhancing patients’ self-management capacity [3].

Although hemodialysis is a life-sustaining therapy, it alone is insufficient to adequately control electrolyte balance and remove metabolic waste, making strict dietary management essential [4]. Dietary adherence in hemodialysis patients is closely associated with key physiological indicators, including interdialytic weight gain (IDWG), hemoglobin (Hb), total protein (TP), albumin (Alb), serum potassium (K), calcium (Ca), and phosphorus (P) [5,6]. However, despite receiving dietary education, many hemodialysis patients forget the information over time or struggle to maintain appropriate dietary practices. This suggests that single-session education has limitations in promoting long-term behavioral change [7]. Although these challenges affect many patients undergoing hemodialysis, they may be particularly pronounced among older adults and individuals with low health literacy. Patients with limited health literacy often experience difficulty understanding complex dietary recommendations and translating such knowledge into sustained dietary practices [3,8].

Spaced repetition, a learning strategy involving repeated exposure to information at regular intervals, has been shown to strengthen long-term memory retention and delay forgetting [9]. Previous studies have demonstrated that repetitive dietary education significantly improves dietary knowledge and self-care adherence in hemodialysis patients [3,6]. Such an approach may be particularly beneficial for patients who experience difficulty retaining or applying dietary information in daily life.

Although older adults constitute a large proportion of patients undergoing hemodialysis, the challenges associated with dietary management are not limited to this group. Patients across various adult age groups, including younger adults undergoing long-term dialysis therapy, must continuously adhere to complex dietary restrictions in order to maintain physiological stability [4]. Therefore, repetitive dietary education may be beneficial not only for older adults but also for younger patients who require sustained support to maintain appropriate dietary practices during long-term treatment [3,8]. These needs may be further amplified in rural areas, which are frequently characterized as medically underserved due to population aging, shortages of healthcare professionals, and limited access to healthcare services, thereby restricting the provision of regular and standardized patient education [10]. Moreover, rural populations generally exhibit lower access to health information and lower levels of health literacy than urban populations [11,12], which may further hinder effective dietary self-management among patients undergoing hemodialysis.

Recent advances in digital-based repetitive learning have emerged as a promising strategy to address existing challenges in hemodialysis patient education. In particular, repetitive education incorporating digital-based content offers the dual advantages of engaging both visual and auditory sensory channels [3,13]. This multimodal stimulation, delivered repeatedly, enhances information retention more effectively than traditional text-based educational materials [13].

The intervention was designed for adult hemodialysis patients in general, although it may be particularly beneficial for older adults and individuals with low health literacy. In response to these challenges, this study implemented a digital repetitive dietary education (DRDE) program developed using official educational videos provided by KSN [14] and evaluated its effectiveness by examining changes in physiological indicators. Furthermore, this study was designed as a pilot investigation to support the development and validation of a tailored DRDE program for hemodialysis patients in rural settings, and its findings are expected to provide foundational evidence for future large-scale intervention studies.

### 2. Purpose of the study

The purpose of this study was to examine the effects of the DRDE program utilizing official educational videos from KSN [14] on dietary knowledge, dietary self-care behaviors, and physiological indicators among hemodialysis patients living in a rural area.

### 3. Research hypotheses

The hypotheses of this study were as follows:

1) Patients in the experimental group receiving the DRDE intervention will demonstrate significantly higher levels of dietary knowledge than those in the control group.

2) Patients in the experimental group receiving the DRDE intervention will demonstrate significantly higher levels of dietary self-care practice behaviors than those in the control group.

3) Patients in the experimental group receiving the DRDE intervention will exhibit significant changes in physiological indicators (IDWG, Hb, TP, Alb, K, Ca, and P) compared with those in the control group.

## METHODS

### 1. Study design

This study employed a quasi-experimental design using a non-equivalent control group pretest-posttest with repeated measures. The DRDE program, reconstructed by the researcher based on official educational videos provided by KSN [14], was applied to patients undergoing hemodialysis. Changes in dietary knowledge, dietary self-care practice behaviors, and physiological indicators were analyzed to evaluate the effects of the intervention. This study was reported in accordance with the TREND (Transparent Reporting of Evaluations with Nonrandomized Designs) statement for nonrandomized intervention studies.

### 2. Participants

The participants were outpatients undergoing maintenance hemodialysis at the hemodialysis unit of Goesan Seongmo Hospital, located in Goesan-gun, Chungcheongbuk-do, South Korea. Individuals who met the study criteria and voluntarily agreed to participate after receiving a full explanation of the study purpose and procedures were included.

The inclusion criteria were as follows:

1) Adult patients (≥ 19 years) undergoing maintenance hemodialysis at the hemodialysis unit of Goesan Seongmo Hospital, located in Goesan-gun, Chungcheongbuk-do, South Korea.

2) Patients receiving hemodialysis 3 times per week for at least 3 months

3) Individuals without visual or hearing impairments and able to read and write

4) Individuals with sufficient cognitive ability to understand and judge the DRDE program (Mini-Cog score ≥ 3)

5) Individuals who voluntarily provided written informed consent after fully understanding the study purpose and procedures

The required sample size was calculated based on the study by Kim and Choi [3], using a power (1−β) of .80, a medium effect size (f) of .25, and a significance level (α) of .05. The analysis indicated that 17 participants per group were required. Considering potential attrition, a total of 40 participants were recruited and assigned to either the experimental group (n = 20) or the control group (n = 20).

### 3. Instruments

#### 1) Dietary knowledge

Dietary knowledge was assessed using a tool developed based on Nutrition and Dietary Management for Hemodialysis Patients, Volume 2, published by KSN [15]. We obtained permission from KSN to use the guideline for research and educational purposes. The instrument consisted of 12 dichotomous (O/X) items covering seven key dietary domains relevant to hemodialysis patients: protein intake, potassium control, P control, sodium restriction, fluid restriction, adherence to phosphate binders, and anemia management. Each item was scored as 1 for a correct response (“O”) and 0 for an incorrect or “do not know” response (“X” or “unknown”), with higher total scores indicating greater dietary knowledge. The clinical practice guidelines of the KSN are educational materials intended to provide clinical recommendations and were not originally developed as a measurement instrument; therefore, their reliability has not been reported. Accordingly, the present study assessed the internal consistency of the dietary knowledge items reconstructed based on the guideline content using KR-20, which resulted in a value of .80.

#### 2) Dietary self-care practice behaviors

Dietary self-care practice behaviors were measured using a tool developed based on the dietary management principles presented in Nutrition and Dietary Management for Hemodialysis Patients, Volume 2 [15]. This behavior-focused instrument was designed to assess whether core dietary knowledge areas were translated into actual daily practices.

Initially, eight preliminary items were generated based on dietary knowledge domains. After consultation with a nephrologist, items were refined to eliminate redundancy and ensure clinical relevance. Content validity was then evaluated by an expert panel consisting of one nephrologist, three dialysis-specialized nurses, and one professor of adult nursing. The scale-level content validity index was .92.

A pilot test was conducted with 10 hemodialysis patients to assess item clarity and response feasibility. The final instrument consisted of five items addressing: (1) food selection during meals, (2) repeated use of educational materials, (3) dietary control when eating out, (4) control of soup and fluid intake, and (5) adherence to potassium- and P-controlling medications. Responses were rated on a 5-point Likert scale (1=strongly disagree to 5=strongly agree), with higher scores indicating better dietary self-care practice. Cronbach’s α for this tool was .88 in the present study.

#### 3) Physiological indicators

Physiological indicators were selected based on the Kidney Disease Outcomes Quality Initiative (KDOQI) clinical practice guidelines [4] to assess nutritional status and electrolyte balance in hemodialysis patients. In consultation with a nephrologist at Goesan Seongmo Hospital, indicators directly related to dietary and fluid management and protein-electrolyte metabolism among routinely measured monthly laboratory tests were initially identified. Subsequently, an expert panel (one nephrologist, three hemodialysis nurses, and one adult nursing professor) reviewed and confirmed the appropriateness of the selected indicators. The final set included seven indicators: IDWG (target 3%–5% of dry weight), Hb (10.0–11.5 g/dL), TP (6.3–8.0 g/dL), Alb (≥ 4.0 g/dL), K (3.5–5.5 mEq/L), Ca (8.4–10.2 mg/dL), and P (3.5–5.5 mg/dL). Laboratory data were obtained from routine tests conducted in the dialysis unit. KDOQI-recommended reference ranges were applied, except for total protein, for which institutional clinical standards were used due to the absence of specific KDOQI criteria.

### 4. Development of the DRDE program

The DRDE program was developed to address the low health literacy and need for repetitive learning among hemodialysis patients in rural areas, as well as practical challenges reported in clinical settings, such as heavy workloads, lack of dedicated educators, and limitations of repeated face-to-face education. To overcome reliance on individualized nurse explanations, the program was designed as a standardized and sustainable educational intervention. The program incorporated principles of spaced repetition based on Ebbinghaus’ Forgetting Curve theory [16]. According to this theory, newly learned information is rapidly forgotten unless it is reinforced through repeated exposure. Therefore, the DRDE program was structured to include an initial review within 2–3 days after learning and re-exposure approximately 7 days later [3]. Previous studies have shown that such repetitive learning structures improve dietary knowledge and self-care adherence among hemodialysis patients [6].

#### 1) Structure and content of the DRDE program

The DRDE program consisted of eight sessions delivered over 4 weeks, with two sessions per week. Educational topics were derived from Nutrition and Dietary Management for Hemodialysis Patients, Volume 2 [15] and aligned with the domains measured by dietary self-care practice behaviors. The detailed session structure and educational content of the DRDE program are presented in Table 1.

Each weekly cycle followed a structured learning pattern:

• Monday: Introduction of a new topic through theory-based video education

• Wednesday: Review using short-form videos and O/X quiz-based reinforcement

• Following Monday: Review of the previous week’s content through Q&A and feedback, followed by the introduction of a new topic

#### 2) DRDE development process

A needs assessment interview was conducted with five hemodialysis patients to identify key educational demands, including difficulty understanding medical terminology, limited use of educational materials, and challenges in dietary control when eating out. Based on these findings, educational content was developed using evidence on the effectiveness of video-based education [17,18], learning strategies appropriate for older adults [3], and principles of multimedia learning [9]. Official educational videos from KSN [14] were utilized, with adjustments made to quiz difficulty and video playback speed to accommodate older adults’ comprehension levels [11,12]. Prior to implementation, the program content and session structure were reviewed by an expert panel of five members. A pilot application was then conducted to assess clarity and delivery methods, after which the program was finalized. Participants received written materials summarizing the program schedule, session topics, learning methods, and expected outcomes.

#### 3) Content validity and inter-educator reliability of the DRDE program

The content validity of the DRDE program was confirmed by an expert panel, with all content validity indices exceeding 0.80. To ensure consistency and standardization in DRDE education, three nurses from Goesan Seongmo Hospital were selected as educators: one hemodialysis nurse with over 10 years of clinical experience (the researcher) and two dialysis nurses. Prior to program implementation, the researcher conducted training and standardization sessions for all educators to promote uniform delivery of the intervention. An educator checklist was used to monitor adherence to the educational protocol and to ensure consistency in instructional delivery across sessions. These procedures established inter-educator reliability and enhanced structural fidelity, ensuring that the DRDE program was implemented consistently and uniformly throughout all sessions.

### 5. Study procedure

**Figure 1.**
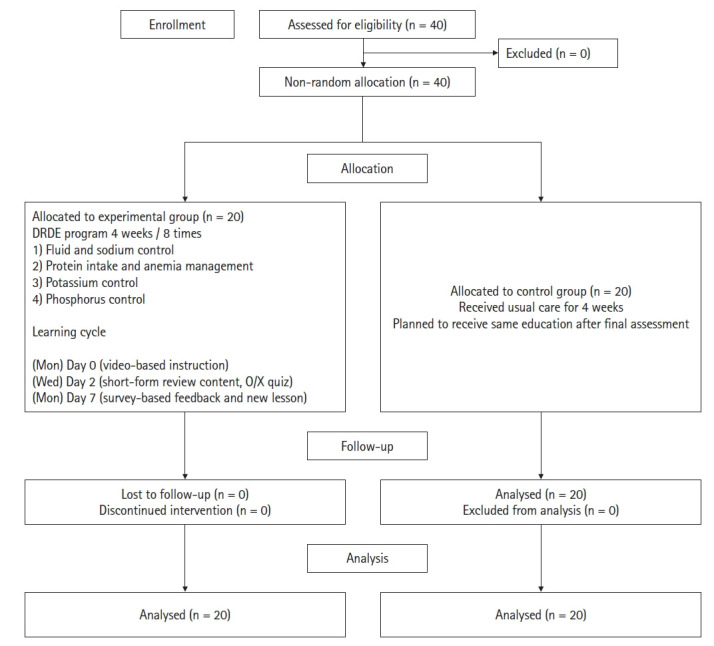
Flow diagram of participant enrollment, allocation, follow-up, and analysis. DRDE = Digital repetitive dietary education; Mon = Monday; Wed = Wednesday.

Approval for study implementation was obtained from the nephrology department, nursing administration, and hospital authorities of Goesan Seongmo Hospital prior to data collection. A convenience sample of 40 patients receiving maintenance hemodialysis 3 times per week for at least 3 months was recruited. Data collection was conducted between July and August 2025. To prevent contamination effects, patients receiving dialysis on Monday-Wednesday-Friday were assigned to the experimental group (n = 20), whereas those receiving dialysis on Tuesday-Thursday-Saturday were assigned to the control group (n = 20) (Figure 1).

#### 1) Pretest

Baseline data on demographic characteristics, disease-related characteristics, dietary knowledge, and dietary self-care practice behaviors were collected using self-administered questionnaires. Physiological indicators were obtained from electronic medical records. Pretest data collection was conducted by the researcher and two trained nurses. Standardized instructions were provided to ensure consistency in questionnaire administration.

#### 2) Intervention

The experimental group received the 8-session DRDE program over 4 weeks. Educational videos were delivered using a laptop during or after dialysis sessions, and participants were also allowed to review the videos on their smartphones as needed. Each session consisted of approximately 20 minutes of video viewing followed by 10 minutes of Q&A. Educator checklists were used to verify delivery of core content, video viewing, Q&A, and feedback. The control group continued to receive routine care, which consisted of brief dietary counseling provided by healthcare professionals during regular laboratory result consultations. After study completion, the control group was offered the same DRDE program to ensure educational equity.

#### 3) Posttest

Posttest assessments were conducted 1 week after the program completion by the researcher and two trained nurses. Dietary knowledge and dietary self-care practice behaviors were measured using the same instruments as at baseline. Physiological indicators were collected from electronic medical records one month after completion of the intervention. All data were anonymized and used solely for research purposes.

### 6. Ethical considerations

This study was approved by the Institutional Review Board of Jungwon University (IRB No. 1044297-HR-202505-003-02). All participants received detailed explanations regarding the study purpose, procedures, voluntary participation, right to withdraw, and confidentiality, and provided written informed consent. Data were managed using de-identified codes and used exclusively for research purposes. After the mandatory 3-year data retention period, all data will be destroyed. Participants in both groups received a small token of appreciation, and the control group was provided with the DRDE program after study completion.

### 7. Data analysis

Data were analyzed using SPSS version 22.0 (IBM Corp., Armonk, NY, USA); 1) Descriptive statistics (frequency and percentage) were used to summarize participants’ general and disease-related characteristics. 2) Normality of dependent variables was assessed using the Kolmogorov–Smirnov test. 3) Baseline homogeneity between the experimental and control groups was examined using the *χ*^2^ test, independent t-test, and Fisher’s exact test. 4) The effects of the DRDE program were analyzed using a 2 × 2 repeated-measures ANOVA to examine the main effects of group (experimental vs. control), time (pretest vs. posttest), and the interaction between group and time, as recommended in statistical literature and previous quasi-experimental intervention studies [19–21].

### 8. Rigor

To enhance methodological rigor and transparency, this study followed the Transparent Reporting of Evaluations with Nonrandomized Designs (TREND) checklist [22]. All relevant TREND items were systematically reviewed and applied throughout the research process. Furthermore, the DRDE program was theoretically grounded in the principle of spaced repetition, derived from Ebbinghaus’ Forgetting Curve theory [16]. The digital video-based educational content was developed based on the study of Yasari et al. [13], and official educational videos provided by KSN [14], thereby ensuring both content validity and educational rigor.

## RESULTS

No adverse events or unintended effects related to the intervention were observed in either study condition.

### 1. Homogeneity test of general characteristics

No statistically significant differences were found between the experimental and control groups in general characteristics, including sex, age, education level, employment status, cause of renal failure, living arrangement, and prior experience with dietary education (all *p* > .050), indicating that the two groups were homogeneous (Table 2).

### 2. Baseline homogeneity of dependent variables

Baseline comparisons of dietary knowledge, dietary self-care practice behaviors, and physiological indicators showed no statistically significant differences between the two groups (all *p* > .050), confirming baseline homogeneity of the dependent variables (Table 3).

### 3. Effects of the DRDE program

Dietary knowledge showed significant effects of time (F = 35.42, *p* < .001), group (F = 10.22, *p* = .003), and the time-by-group interaction (F = 14.83, *p* = .001). Dietary self-care practice behaviors showed a significant effect of time (F = 7.56, *p* = .009) and a significant time-by-group interaction (F = 4.21, *p* = .048), whereas the main effect of group was not significant (F = 0.88, *p* = .354).

Among physiological indicators, IDWG showed significant effects of time (F = 8.94, *p* = .005) and the time-by-group interaction (F = 7.02, *p* = .012). K showed significant effects of time (F = 11.78, *p* = .002) and the time-by-group interaction (F = 9.55, *p* = .004). Serum P also showed significant effects of time (F = 5.14, *p* = .030) and the time-by-group interaction (F = 4.62, *p* = .038). In contrast, Hb, TP, Alb, and Ca showed no significant effects of time, group, or the time-by-group interaction (all *p* > .050) (Table 4).

## DISCUSSION

This study implemented the DRDE program reconstructed by the researcher based on official educational materials provided by KSN to rural hemodialysis patients and examined changes in dietary knowledge, dietary self-care practice behaviors, and physiological indicators. Dietary knowledge differed significantly by time, group, and the time-by-group interaction, indicating that both groups changed over time, that overall levels differed between groups, and that the magnitude of change across time varied by group. Notably, the experimental group demonstrated a greater increase in dietary knowledge than the control group, suggesting that DRDE produced a stronger knowledge gain in the intervention group.

This finding is consistent with previous studies reporting that repetitive dietary education is effective in improving dietary knowledge among hemodialysis patients [3,5,8,17,23]. In particular, Kim and Choi [3], Park and Choi [8], and Kim and Yoo [23] reported that repetitive, systematic education-rather than single-session education-enhances comprehension of dietary information and supports long-term memory formation. Similarly, the present intervention repeatedly exposed participants to the same content at planned intervals while using audiovisual materials, thereby complementing the limitations of one-time verbal education and promoting both understanding and retention of dietary knowledge. This approach can be interpreted as an intervention strategy grounded in spaced repetition and multimedia learning principles that account for forgetting over time [9,16].

Although the intervention period was relatively short, the significant effects observed for time, group, and the time-by-group interaction support the feasibility of sustained application of DRDE. Accordingly, DRDE may be expanded into a long-term repetitive education system and standardized as part of routine education for hemodialysis patients; moreover, providing the same education to the control group after study completion may ethically facilitate dissemination of the educational benefits to the broader patient population. Standardizing educational content and procedures may also enable all nurses to deliver patient education according to consistent criteria, thereby reducing between-group disparities and strengthening dietary self-management capacity.

Dietary self-care practice behaviors showed significant effects of time and the time-by-group interaction, whereas the main effect of group was not significant. This finding suggests that dietary practice behaviors may change gradually through repeated reinforcement over time and adaptation to individual living contexts after knowledge acquisition. Previous studies have also reported that improvements in dietary practice behaviors tend to emerge progressively after a certain period rather than immediately after education [5,8,24,25]. Therefore, the absence of a clear main group effect in the short-term intervention aligns with prior evidence. To improve dietary practice behaviors, interventions should extend beyond short-term education and incorporate long-term, repetitive approaches that continuously reinforce behavioral change. Because dietary practices are closely related to food choices made in the home environment outside the dialysis unit, education should involve not only patients but also family caregivers [26]. Caregiver education may facilitate shared understanding of dietary management and promote a supportive home environment, thereby contributing to sustained and stable dietary self-care practice behaviors [27,28]. In clinical practice, healthcare professionals can actively involve caregivers in the educational process to strengthen patients’ dietary adherence. For example, caregivers may be encouraged to accompany patients during dialysis sessions to receive dietary education together, and additional support may be provided through telephone-based counseling. Furthermore, sharing educational video materials may allow caregivers to review dietary management strategies at home and support repeated learning, thereby reinforcing patients’ dietary self-care behaviors.

Regarding physiological indicators, IDWG, serum K, and serum P showed significant effects of time and time-by-group interaction, suggesting that the DRDE program may have influenced outcomes related to dietary and fluid control. Since hemodialysis primarily functions to remove accumulated metabolic waste and maintain electrolyte balance in patients with renal failure, effective dietary management between dialysis sessions is essential for preventing excessive accumulation of potassium and P. In particular, reductions in serum K and P may reflect improved understanding and implementation of dietary control through repetitive education on electrolyte content of foods, cooking and intake strategies, and adherence to phosphate binders. These findings are consistent with previous studies reporting that structured dietary education improves dietary management behaviors and contributes to favorable changes in biochemical indicators among hemodialysis patients [4,8,17]. Kim and Choi [3] and Park and Choi [8] reported that repetitive dietary education programs improved dietary management and were associated with favorable changes in biochemical indicators among hemodialysis patients. Similarly, Pack and Lee [29] demonstrated that a smartphone application–based dietary self-management program improved dietary management behaviors and contributed to improvements in clinical indicators. In contrast, Alb, Hb, and Ca did not show significant changes after the short-term intervention. This may be attributable to the fact that these indicators are influenced by multiple factors, including longer-term nutritional status, inflammation, erythropoietic function, and pharmacologic treatment [4]. Therefore, future studies should extend the intervention duration and/or employ follow-up assessments to evaluate the sustainability and clinical significance of physiological changes.

The present study provides preliminary evidence regarding the feasibility and potential effectiveness of a DRDE program for hemodialysis patients. Although the intervention period was relatively short, improvements in key physiological indicators such as serum potassium, P, and IDWG suggest that structured and repetitive dietary education may positively influence electrolyte and fluid management. In addition, future research should comprehensively examine how changes in physiological indicators relate to self-management capacity and quality of life among hemodialysis patients. Prior research has reported that higher self-management is significantly associated with better quality of life in hemodialysis patients, implying that strengthening self-management through repetitive dietary education may translate into long-term improvements in quality of life [30].

During implementation in this study, some participants demonstrated limited awareness of the cause of their disease and did not perceive explanations of laboratory results provided by healthcare professionals as “education.” This observation suggests that rural hemodialysis patients may face constrained access to medical information and low health literacy, consistent with previous studies reporting health information disparities and insufficient digital health literacy among rural older adults [11,12,31]. Such characteristics may act as key barriers in translating dietary knowledge into actual behaviors, supporting the need for repetitive education tailored to patients’ comprehension levels. Moreover, regional disparities in nursing workforce distribution may constitute a structural barrier to providing systematic and continuous patient education in rural healthcare institutions. Park and Kim [32] argued that this workforce imbalance cannot be resolved solely through increasing staffing numbers and highlighted the need to improve working conditions and develop region-specific workforce utilization strategies. These structural constraints may contribute to one-time or non-standardized patient education and may undermine the quality and continuity of education.

In this context, the DRDE program applied in the present study may be considered an intervention designed to deliver relatively consistent and standardized education despite limitations of rural healthcare environments. The program was based on official educational videos and dietary guidelines provided by KSN, supporting the credibility and clinical validity of the content [14,15]. By adopting a digital repetitive structure, DRDE aimed to enhance both retention of dietary knowledge and translation into behavioral practice. Accordingly, DRDE may serve as a feasible nursing intervention strategy to strengthen dietary management capacity among rural hemodialysis patients [29,33].

## CONCLUSION

This study aimed to evaluate the effectiveness of the DRDE program among hemodialysis patients in a rural area of South Korea. The experimental group demonstrated significantly greater improvement in dietary knowledge than the control group, and dietary self-care practice behaviors showed a gradual improvement over time. In addition, K and P levels decreased significantly, suggesting that a structured, repetitive, audiovisual-based education program can positively influence dietary management and electrolyte control in hemodialysis patients.

A key strength of this study lies in the development of the DRDE program based on standardized educational materials provided by the KSN, ensuring consistency and accuracy of content. The use of digital and audiovisual media enabled repeated learning without requiring additional face-to-face instruction, thereby reducing the educational burden on nursing staff. From a nursing practice perspective, this approach supports the feasibility of implementing continuous and systematic dietary education in rural and primary care settings, where staffing and educational resources are often limited. Furthermore, by overcoming the limitations of non-systematic, single-session education, the DRDE program may support understanding of dietary management and disease-related self-care among rural hemodialysis patient, highlighting its practical value as a nursing intervention.

Nevertheless, several limitations should be considered when interpreting the findings. The study was conducted over a relatively short intervention period with a small sample size and was limited to a single primary healthcare institution in one rural area, which may restrict the generalizability of the results. Accordingly, future multicenter studies involving larger samples across diverse regions and healthcare settings are recommended to validate the effectiveness of the DRDE program. In addition, long-term follow-up studies of at least six months are needed to examine the sustained effects of the intervention on dietary self-care behaviors and physiological indicators.

Future research should also focus on developing tailored digital educational content and mobile-based review systems that reflect patients’ cognitive levels, learning capacities, and individual needs. Such refinements may further enhance patient engagement, self-management capacity, and the overall effectiveness of digital education programs. Ultimately, the DRDE program has the potential to function as a sustainable, community-based nursing intervention that strengthens self-care among rural hemodialysis patients while simultaneously alleviating the workload burden of nursing staff.

## Figures and Tables

**Table 1.** Structure and Educational Content of the Digital Repetitive Dietary Education (DRDE) Program

Week	Session	Topic	Learning activities	Duration (minutes)
1	1 (Mon)	Fluid and sodium control (IDWG)	• Introduction: Orientation and goal setting for fluid and sodium control	30
			• Content: Video-based instruction on salt restriction and weight monitoring	
			• Summary: Key point review and Q&A	
	2 (Wed)	Review and reinforcement	• Content: Short-form review video on practical low-sodium cooking tips	30
			• Summary: O/X quiz (Q1, Q2) for knowledge reinforcement and feedback	
2	3 (Mon)	Protein intake and Anemia management	• Introduction: Feedback on Week 1 adherence and review of survey responses	30
			• Content: Video-based instruction on protein selection and iron supplements	
			• Summary: Review of strategies for maintaining appropriate serum albumin levels	
	4 (Wed)	Review and reinforcement	• Content: Short-form review video on high-quality protein intake	30
			• Summary: True/false quiz (Q3, Q4) for knowledge reinforcement and feedback	
3	5 (Mon)	Potassium control	• Introduction: Feedback on Week 2 adherence and review of survey responses	30
			• Content: Video-based instruction on identifying high-potassium foods and potassium-leaching methods	
			• Summary: Discussion of safe food alternatives for rural dialysis patients	
	6 (Wed)	Review and reinforcement	• Content: Short-form review video on effective potassium reduction techniques	30
			• Summary: True/false quiz (Q5, Q6) for knowledge reinforcement and feedback	
4	7 (Mon)	Phosphorus control	• Introduction: Feedback on Week 3 adherence and review of survey responses	30
			• Content: Video-based instruction on proper use of phosphate binders and interpretation of food labels	
			• Summary: Review of dining-out strategies and hidden sources of phosphorus	
	8 (Wed)	Final review and reinforcement	• Content: Comprehensive review video covering all core dietary management domains	30
			• Summary: Final True/false quiz (Q7, Q8), motivation for sustained self-care, and program closure	

The DRDE program consisted of eight sessions delivered over 4 weeks (two sessions per week). Each session followed a structured format including an introduction, content delivery through video-based education, and a summary phase involving review or quiz-based reinforcement. IDWG = Interdialytic weight gain; Mon = Monday; Wed = Wednesday; Q = question.

**Table 2.** Homogeneity Testing of General and Disease-related Characteristics (N = 40)

Characteristics	Classification	Exp. (n = 20)	Cont. (n = 20)	χ² or t	*p*
Sex	Men	11 (55)	11 (55)	0.00	1.000
	Women	9 (45)	9 (45)		
Age (years)	< 65	8 (40)	11 (55)	0.90	.342
	≥ 65	12 (60)	9 (45)		
Education^†^	≤ Middle school	8 (40)	7 (35)	0.27	.875
	High school	10 (50)	10 (50)		
	≥ College	2 (10)	3 (15)		
Living status^†^	Alone	3 (15)	8 (40)	3.14	.155
	With family	17 (85)	12 (60)		
Occupation^†^	Employed	5 (25)	4 (20)	0.14	1.000
	Unemployed	15 (75)	16 (80)		
Cause of renal failure^†^	Hypertension	6 (30)	3 (15)	1.81	.765
	Diabetes	9 (45)	9 (45)		
	Glomerulonephritis	1 (5)	2 (10)		
	Others	1 (5)	2 (10)		
	Unknown	3 (15)	4 (20)		
Duration of dialysis^†^ (years)	< 3	7 (35)	9 (45)	0.65	.721
	3–< 7	6 (30)	4 (20)		
	≥ 7	7 (35)	7 (35)		
Experience of DE	Yes	17 (85)	16 (80)	0.17	1.000
	No	3 (15)	4 (20)		
Type of DE^†^	None	3 (15)	4 (20)	3.28	.109
	Face-to-face	14 (70)	16 (80)		
	Printed material	3 (15)	0 (0)		
Source of DI	Medical staff	15 (75)	18 (90)	4.27	.140
	Caregiver	0 (0)	1 (5)		
	TV/Internet	3 (15)	1 (5)		
	None	2 (10)	0 (0)		
Perceived importance of DM^†^	Very important	14 (70)	11 (55)	1.65	.362
	Important	6 (30)	8 (40)		
	Not important	0 (0)	1 (5)		
Difficulties in DM^†^	Many food restrictions	8 (40)	9 (45)	7.86	.062
	Lifelong restriction	3 (15)	1 (5)		
	Lack of knowledge	4 (20)	1 (5)		
	Frequent eating out	2 (10)	0 (0)		
	Hard to practice	3 (15)	9 (45)		
Usefulness of online education^†^	Very helpful	2 (10)	3 (15)	0.96	.810
	Somewhat helpful	14 (70)	11 (55)		
	Not helpful	2 (10)	3 (15)		
	Not helpful at all	2 (10)	3 (15)		

Values are presented as n (%). Exp. = Experimental group; Cont. = Control group; DE = Dietary education; DI = Dietary information; DM = Dietary management.

† Fisher’s exact.

**Table 3.** Homogeneity Testing of Dependent Variables (N = 40)

Variables	Exp. (n = 20)	Cont. (n = 20)	t	*p*
Pre-dietary knowledge score (12 points)	7.70 ± 2.64	7.60 ± 1.79	0.14	.889
Dietary behavior (25 points)	18.63 ± 4.36	18.43 ± 3.44	0.16	.874
Interdialytic weight gain (kg)	2.60 ± 1.19	2.72 ± 1.35	-0.30	.767
Hemoglobin (g/dL)	10.30 ± 1.15	10.19 ± 0.66	-0.39	.700
Total protein (g/dL)	6.86 ± 0.43	6.82 ± 0.31	0.30	.769
Albumin (g/dL)	4.16 ± 0.33	4.17 ± 0.25	-0.05	.958
Potassium (mEq/L)	5.57 ± 0.91	5.38 ± 0.81	0.70	.490
Calcium (mg/dL)	9.51 ± 1.22	9.35 ± 1.02	0.44	.665
Phosphorus (mg/dL)	5.04 ± 1.74	4.87 ± 1.23	0.35	.729

Values are presented as mean ± standard deviation. Exp. = Experimental group; Cont. = Control group.

**Table 4.** Effects of the DRDE Program on Dependent Variables (N = 40)

Variables	Group	Pretest	Posttest	Group	Time	Group × Time F (*p*)
				F (*p*)	F (*p*)	
Dietary knowledge	Exp.	7.70 ± 2.64	9.95 ± 1.43	10.22 (.003)	35.42 (< .001)	14.83 (.001)
	Cont.	7.60 ± 1.79	7.90 ± 1.68			
Dietary behavior	Exp.	18.63 ± 4.36	20.58 ± 3.15	0.88 (.354)	7.56 (.009)	4.21 (.048)
	Cont.	18.43 ± 3.44	19.38 ± 3.31			
IDWG (kg)	Exp.	2.60 ± 1.19	2.06 ± 0.83	2.13 (.153)	8.94 (.005)	7.02 (.012)
	Cont.	2.72 ± 1.35	2.93 ± 1.11			
Hemoglobin (g/dL)	Exp.	10.30 ± 1.15	10.58 ± 1.19	0.33 (.568)	1.28 (.265)	0.52 (.475)
	Cont.	10.19 ± 0.66	10.28 ± 0.70			
Total protein (g/dL)	Exp.	6.86 ± 0.43	6.78 ± 0.43	1.62 (.211)	0.92 (.344)	0.41 (.524)
	Cont.	6.82 ± 0.31	6.53 ± 0.35			
Albumin (g/dL)	Exp.	4.16 ± 0.33	4.13 ± 0.21	0.57 (.454)	2.04 (.162)	0.36 (.553)
	Cont.	4.17 ± 0.25	4.08 ± 0.28			
Potassium (mEq/L)	Exp.	5.57 ± 0.91	4.83 ± 0.61	1.83 (.184)	11.78 (.002)	9.55 (.004)
	Cont.	5.38 ± 0.81	5.42 ± 0.75			
Calcium (mg/dL)	Exp.	9.51 ± 1.22	9.62 ± 1.14	0.41 (.524)	0.87 (.357)	0.59 (.448)
	Cont.	9.35 ± 1.02	9.46 ± 0.89			
Phosphorus (mg/dL)	Exp.	5.04 ± 1.74	4.52 ± 1.14	0.76 (.389)	5.14 (.030)	4.62 (.038)
	Cont.	4.87 ± 1.23	4.65 ± 1.20			

Values are presented as mean ± standard deviation. DRDE = Digital repetitive dietary education; Exp. = Experimental; Cont. = Control; IDWG = Interdialytic weight gain.
